# Loss of Fhit expression in non-small-cell lung cancer: correlation with molecular genetic abnormalities and clinicopathological features

**DOI:** 10.1054/bjoc.1999.1062

**Published:** 2000-02-21

**Authors:** J Geradts, K M Fong, P V Zimmerman, J D Minna

**Affiliations:** Nuffield Department of Pathology and Bacteriology, University of Oxford, John Radcliffe Hospital, Academic Block, Level 4, Oxford, OX3 9DU, UK; Department of Thoracic Oncology, The Prince Charles Hospital, Brisbane, Queensland, 4032, Australia; Hamon Center for Therapeutic Oncology Research, University of Texas Southwestern Medical Center, Dallas, TX, 75390, USA

**Keywords:** FHIT, immunohistochemistry, lung cancer, prognosis, loss of heterozygosity

## Abstract

The *FHIT* gene is located at a chromosomal site (3p14.2) which is commonly affected by translocations and deletions in human neoplasia. Although *FHIT* alterations at the DNA and RNA level are frequent in many types of tumours, the biological and clinical significance of these changes is not clear. In this study we aimed at correlating loss of Fhit protein expression with a large number of molecular genetic and clinical parameters in a well-characterized cohort of non-small-cell lung cancers (NSCLCs). Paraffin sections of 99 non-small-cell carcinomas were reacted with an anti-Fhit polyclonal antibody in a standard immunohistochemical reaction. Abnormal cases were characterized by complete loss of cytoplasmic Fhit staining. The Fhit staining results were then correlated with previously obtained clinical and molecular data. Fifty-two of 99 tumours lacked cytoplasmic Fhit staining, with preserved reactivity in adjacent normal cells. Lack of Fhit staining correlated with: loss of heterozygosity (LOH) at the *FHIT* 3p14.2 locus, but not at other loci on 3p; squamous histology; LOH at 17p13 and 5q but not with LOH at multiple other suspected tumour suppressor gene loci; and was inversely correlated with codon 12 mutations in K- *ras*. Fhit expression was not correlated overall with a variety of clinical parameters including survival and was not associated with abnormalities of immunohistochemical expression of p53, RB, and p16. All of these findings are consistent with loss of Fhit protein expression being as frequent an abnormality in lung cancer pathogenesis as are p53 and p16 protein abnormalities and that such loss occurs independently of the commitment to the metastatic state and of most other molecular abnormalities. © 2000 Cancer Research Campaign


*British Journal of Cancer* (2000) **82**(6), 1191–1197
© 2000 Cancer Research Campaign
Article no. bjoc.1999.1062, available online at http://www.idealibrary.com on IDEAL®

### Loss of Fhit expression in non-small-cell lung cancer: correlation with molecular genetic abnormalities and clinicopathological features

**J Geradts[1]*, KM Fong[2]*, PV Zimmerman[2] and JD Minna[3]**

[1]Nuffield Department of Pathology and Bacteriology, University of Oxford, John Radcliffe Hospital, Academic Block, Level 4, Oxford OX3 9DU, UK; [2]Department of Thoracic Oncology, The Prince Charles Hospital, Brisbane, Queensland 4032, Australia; [3]Hamon Center for Therapeutic Oncology Research, University of Texas Southwestern Medical Center, Dallas, TX 75390, USA

**Summary** The *FHIT* gene is located at a chromosomal site (3p14.2) which is commonly affected by translocations and deletions in human neoplasia. Although *FHIT* alterations at the DNA and RNA level are frequent in many types of tumours, the biological and clinical significance of these changes is not clear. In this study we aimed at correlating loss of Fhit protein expression with a large number of molecular genetic and clinical parameters in a well-characterized cohort of non-small-cell lung cancers (NSCLCs). Paraffin sections of 99 non-small-cell carcinomas were reacted with an anti-Fhit polyclonal antibody in a standard immunohistochemical reaction. Abnormal cases were characterized by complete loss of cytoplasmic Fhit staining. The Fhit staining results were then correlated with previously obtained clinical and molecular data. Fifty-two of 99 tumours lacked cytoplasmic Fhit staining, with preserved reactivity in adjacent normal cells. Lack of Fhit staining correlated with: loss of heterozygosity (LOH) at the *FHIT* 3p14.2 locus, but not at other loci on 3p; squamous histology; LOH at 17p13 and 5q but not with LOH at multiple other suspected tumour suppressor gene loci; and was inversely correlated with codon 12 mutations in K-*ras*. Fhit expression was not correlated overall with a variety of clinical parameters including survival and was not associated with abnormalities of immunohistochemical expression of p53, RB, and p16. All of these findings are consistent with loss of Fhit protein expression being as frequent an abnormality in lung cancer pathogenesis as are p53 and p16 protein abnormalities and that such loss occurs independently of the commitment to the metastatic state and of most other molecular abnormalities. © 2000 Cancer Research Campaign

**Keywords**: FHIT; immunohistochemistry; lung cancer; prognosis; loss of heterozygosity

Lung cancer is the commonest cause of cancer deaths in Western countries. It is generally believed that cancer is the end-result of a multistep process involving the activation of dominant oncogenes and the inactivation of tumour suppressor genes. In 1996, the Fragile Histidine Triad (*FHIT*) gene was cloned and shown to reside in FRA3B at chromosome band 3p14.2, the most active of the common fragile sites in the human genome (Ohta et al, 1996). This is a very large locus which also encompasses a hereditary renal carcinoma associated chromosome translocation breakpoint. *FHIT* alterations are frequent in a variety of solid tumours and cancer cell lines, including lung cancer (Ohta et al, 1996; Sozzi et al, 1996; Fong et al, 1997). We and others have previously described a high frequency of allelic deletion and abnormal mRNA transcripts in primary lung cancers and cell lines derived from lung tumours (Sozzi et al, 1996; Fong et al, 1997). However, the *FHIT* alterations in lung cancers have demonstrated features not usually attributed to classical tumour suppressor genes such as a lack of point mutations and the presence of associated wild-type transcripts, prompting a number of investigators to query the role of *FHIT* as a suppressor gene. Nevertheless, transfection of wild-type *FHIT* into lung and other cancers with *FHIT* abnormalities results in tumour suppression in vivo (Siprashvili et al, 1997). Recently we have confirmed this tumour suppressor effect in lung cancer (Ji et al, submitted) although Otterson et al (1998) presented results suggesting *FHIT* does not suppress tumourgenicity.

Recent immunohistochemical studies have shown that loss of Fhit expression occurs in a high proportion of primary lung carcinomas and precancerous lesions, a feature consistent with function as a tumour suppressor (Sozzi et al, 1998; Tomizawa et al, 1998). *FHIT* inactivation appears particularly important in the squamous cell carcinoma subtype of lung cancers, and may be related to smoking history (Sozzi et al, 1997*a*, 1998). Fhit protein may also be absent or reduced in gastric, renal, pancreatic, and cervical carcinomas (Greenspan et al, 1997; Baffa et al, 1998; Hadaczek et al, 1998; Simon et al, 1998). Immunohistochemical detection has the advantage of detecting protein loss regardless of the underlying mechanism and thus represents an efficient method of identifying functional protein inactivation. In this report, we studied Fhit protein expression in a spectrum of non-small-cell lung cancers by immunohistochemistry (IHC) and correlated these findings with detailed clinicopathological features including tumour stage, histological tumour type, smoking history and survival. A significant advantage of our cohort is that these primary lung tumours have been extensively investigated for other somatic mutational events including 3p allele loss, particularly in the vicinity of the

*Received 24 May 1999*
*Revised 4 October 1999*
*Accepted 6 October 1999*

*Correspondence to*: J Geradts

*These authors contributed equally to this work

*FHIT* locus, as well as other allele loss at suspected tumour suppressor loci (Fong et al, 1994, 1995*a*, 1995*b*, 1995*c*, 1997). Our aims were to determine whether loss of heterozygosity (LOH) at the *FHIT* locus was associated with loss of protein expression, to determine the prognostic significance of Fhit down-regulation, to confirm the association between Fhit expression and tumour histology, and to probe for correlations with a variety of other genetic abnormalities in this cohort of well characterized NSCLCs.

### MATERIALS AND METHODS

### Patient material

Tumour blocks were obtained from 108 patients with primary NSCLC at The Prince Charles Hospital, Brisbane, Australia who had been treated with curative resectional surgery. This cohort of unselected patients had previously been investigated for molecular genetic changes at candidate chromosomal regions (Fong et al, 1994, 1995*a*, 1995*b*, 1995*c*, 1997). From this cohort, pathology review (by JG) identified 99 cases where adequate material remained for immunohistochemical evaluation. The samples were collected from June 1990 to March 1993 and survival data of 5 or more years were available on most patients. There were 70 males and 29 females, with an age range of 28–81 years (mean age 61 ± 11 years at diagnosis). The patients had an overall 43 ± 32 mean pack years of smoking (range 0–150 pack years). Independent histological examination of the tumours was performed according to 1982 WHO criteria and pathologically confirmed pTNM stage was assigned in accordance with the International Union Against Cancer. There were 56 stage I, 22 stage II and 21 stage III tumours. Histological subtypes included 39 squamous cell carcinomas (SCCs), 43 adenocarcinomas (including six with bronchiolo-alveolar characteristics), 11 adenosquamous cancers, two large-cell carcinomas, three atypical carcinoids and one typical carcinoid. A detailed medical and smoking history was available and there were eight never/non-smokers and 91 smokers with mean pack years of 46 (range 1–150). This study is part of an ongoing research project and thus data on other molecular markers were available for analysis including: K-*ras* codon 12 mutations; *p53* abnormalities; 3p14.2 LOH, LOH at other loci on 3p, and LOH at known or putative tumour suppressor gene loci on 1p, 5q, 8p, 9p, 11p, 13q, 17p, 17q and 18q; and p53, RB and p16 expression by IHC (Fong et al, 1994, 1995*a*, 1995*b*, 1995*c*, 1998; Geradts et al, 1999). As a positive external Fhit control, we used normal kidney. As negative external controls, we used cell blocks prepared from the *FHIT*-deleted lung cancer cell lines NCI-H661 and NCI-H1915 (Fong et al, 1997).

### Immunohistochemistry

One representative block was retrieved for each case, and four-micron paraffin sections were prepared and used within 72 h for immunohistochemistry (see below) or stored at 4°C. The paraffin sections were reacted with rabbit polyclonal anti-Fhit antibody (generously provided by Dr Kay Huebner at the Kimmel Cancer Center in Philadelphia, PA, USA) which had been raised against a glutathione/S-transferase (GST)-Fhit fusion protein (Siprashvili et al, 1997; Sozzi et al, 1997*b*) without a preceding antigen retrieval step. The antibody was used at a 1:2500 dilution for 2 h at room temperature. Non-specific rabbit serum (negative control reaction) was used under identical conditions. The detection reaction followed the Vectastain Elite ABC kit protocol (Vector, Burlingame, CA, USA). Diaminobenzidene was used as chromogen, and haematoxylin as counterstain.

### Interpretation of stains

Fhit-positive cell lines and tissues were characterized by diffuse, moderate to strong cytoplasmic staining. A tumour was considered Fhit-positive if there was cytoplasmic reactivity in the neoplastic cells. The tumour was scored as Fhit-negative if there was no cytoplasmic reactivity within the neoplasm, with preserved reactivity in admixed non-neoplastic cells. Fhit-positive internal controls included columnar epithelium, type II pneumocytes, alveolar macrophages and nerves.

### Statistical analysis

Statistical analysis of correlations between variables was performed using either $\chi^2$ test or Fisher's exact test for categorical data or *t*-test for means. Survival curves were calculated by the Kaplan–Meier method and compared by log rank analysis using SPSS for Windows V8.

### RESULTS

### Fhit expression patterns

The Fhit staining patterns clearly fell into two categories. Forty-seven lung tumours showed cytoplasmic reactivity in the majority of neoplastic cells, usually of moderate to strong intensity. These were considered Fhit-positive (normal expression pattern). A representative Fhit-positive tumour is depicted in Figure 1A. The remaining 52 lung cancers were characterized by complete absence of cytoplasmic staining (abnormal expression pattern). Admixed non-neoplastic elements served as Fhit-positive internal controls (Figure 1B).

### Correlation of Fhit expression with tumour histology

We found a highly significant correlation between Fhit expression and lung cancer histology (Table 1). Thirty-seven of 50 (74%) NSCLCs with squamous differentiation, including 28 of 39 (72%) SCCs and nine of 11 (82%) adenosquamous carcinomas were Fhit-negative. In contrast, only 12 of 47 (25%) tumours with glandular differentiation, including 11 of 38 (27%) adenocarcinomas and one of six bronchiolo-alveolar carcinomas (BACs) (17%), expressed Fhit. Our cohort also included two large cell carcinomas, one of which was Fhit-negative. The single carcinoid tumour was Fhit-positive. In contrast, all three atypical carcinoids had lost Fhit.

### Correlation of Fhit expression with LOH at 3p14.2 and other chromosomal loci and with K-*ras* codon 12 mutations

There was a correlation ($P = 0.007$) between loss of Fhit expression and LOH at 3p14.2, the locus of the *FHIT* gene, as determined by microsatellite analysis of two intragenic markers

 *© 2000 Cancer Research Campaign*

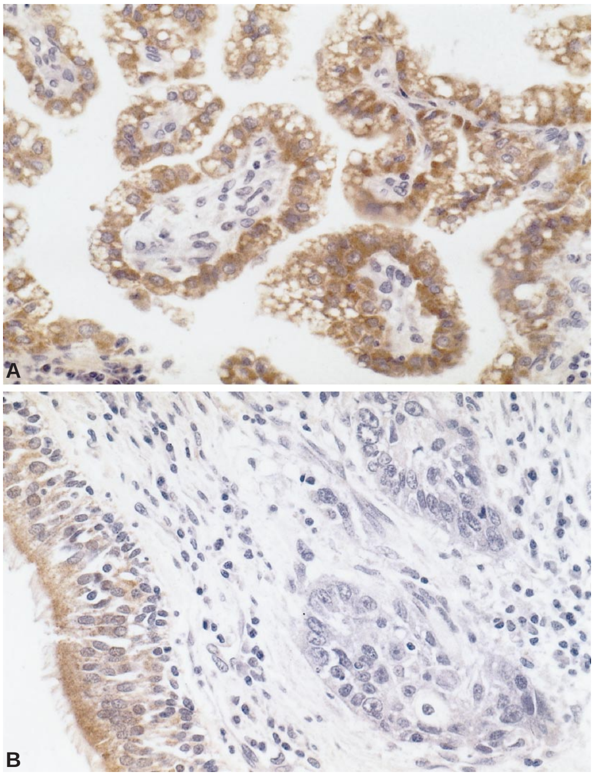

**Figure 1** Fhit expression in NSCLC. (**A**) Fhit-positive papillary adenocarcinoma. Note strong cytoplasmic staining in almost all tumour cells. (**B**) Fhit-negative squamous cell carcinoma. The poorly differentiated tumour nests on the right are entirely negative. The overlying bronchial epithelium (left) serves as an internal positive control. Original magnifications 400×

(Table2). In contrast, Fhit negativity was not associated with LOH at two more distal loci on 3p (Table 2). LOH data were available on nine additional chromosomal arms that are the sites of known or suspected tumour suppressor genes. There was no statistically significant correlation between LOH at seven of these loci and Fhit expression (Table 2). However, loss of Fhit was associated with LOH at the *MCC/APC* locus on 5q ($P$ = 0.006) and with LOH at the p53 locus on 17p13 ($P$ = 0.023, Table 3). Conversely, there was an inverse relationship between Fhit negativity and the presence of a mutation in codon 12 of the K-*ras* gene ($P$ = 0.016, Table 3).

### Correlation of Fhit expression with p53, RB and p16 protein expression abnormalities

The relationship between Fhit and p53, RB and p16 protein expression has previously not been studied in detail. We found no association in this cohort between Fhit immunohistochemical staining status and expression of the three important tumour suppressor genes p53 ($P$ = 0.215), RB ($P$ = 0.511) and p16 ($P$ = 0.96). It has been suggested that there may be a correlation between Fhit and p53 abnormalities in lung cancer (Burke et al, 1998; Marchetti et al, 1998*b*). In this series of NSCLCs, we had previously studied p53 abnormalities by PCR-SSCP (polymerase chain reaction single strand conformation polymorphism analysis) and immunohistochemical staining (Fong et al, 1995*c*; Geradts et al, 1999). While there was a correlation between Fhit negativity and LOH at 17p13, the chromosomal p53 locus, there was no correlation between loss of Fhit expression and mutational analysis of p53 exons 5–8 by PCR-SSCP or p53 overexpression by IHC (Table 3).

### Correlation of Fhit expression with clinical features and survival

In this cohort of 99 NSCLCs there was no statistically significant correlation between loss of Fhit expression and gender, age at presentation, smoking history, tumour size, nodal involvement and stage of disease (Table 4). Likewise, there was no difference in overall survival between patients with Fhit-negative and Fhit-positive tumours (Figure 2).

### DISCUSSION

*FHIT* abnormalities in tumours are often manifest as loss of one allele with abnormal transcripts or deletion of the remaining allele, whereas point mutations are rare (Sozzi et al, 1996; Fong et al, 1997; Nelson et al, 1998). Loss of expression of Fhit, the protein product of the gene, has been described in several common types of human neoplasia including lung cancer (Sozzi et al, 1997*b*, 1998; Tomizawa et al., 1998). Immunohistochemistry is particularly well suited to demonstrate loss of Fhit protein expression in the neoplastic cell population in clinically relevant materials, whatever the underlying mechanism. In our series of 99 Australian NSCLCs, 52 showed complete loss of cytoplasmic Fhit staining with retention of staining in the adjacent non-neoplastic tissues such as bronchial mucosa (Figure 1B). This frequency is between the loss of Fhit protein expression rates previously reported by the Japanese series of Tomizawa et al (34%) (Tomizawa et al, 1998) and the European series of Sozzi et al (73%) (Sozzi et al, 1998). We did not see the intermediate level of Fhit staining, as reported by other groups in 15–20% of lung cancers (Kovatich et al, 1998; Tomizawa et al, 1998) and as may be seen in other types of tumours (Greenspan et al, 1997; Hadaczek et al, 1998). These discrepancies may well be due to differences in immunohistochemical techniques, stain interpretation, or different distributions of histological subtypes (see below).

**Table 1** Correlation between Fhit expression and histological tumour type in 99 non-small cell lung cancers

| | Squamous cell carcinoma | Adenosquamous carcinoma | Adenocarcinoma | Bronchiolo-alveolar carcinoma | Other[a] | Total |
|---|---|---|---|---|---|---|
| Fhit+ | 11 (28%) | 2 (18%) | 27 (73%) | 5 (83%) | 2 (33%) | 47 (47%) |
| Fhit– | 28 (72%) | 9 (82%) | 10 (27%) | 1 (17%) | 4 (67%) | 52 (53%) |
| Total | 39 | 11 | 37 | 6 | 6 | 99 |

$P$ < 0.001. [a]Other = large cell carcinoma ($n$ = 2), carcinoid ($n$ = 1), atypical carcinoid ($n$ = 3).

© 2000 Cancer Research Campaign

**Table 2** Correlation between Fhit expression and LOH at the FHIT locus (3p14.2) and other tumour suppressor gene loci

| Chromosomal locus (microsatellite markers) | | Fhit immunohistochemistry Positive | Fhit immunohistochemistry Negative | *P* ($\chi^2$) |
|---|---|---|---|---|
| 3p14.2 (D3S1300, D3S4103) | LOH (–) | 29 | 20 | 0.007[a] |
| | LOH (+) | 13 | 29 | |
| 3p21 (D3S1029) | LOH (–) | 23 | 20 | 0.784 |
| | LOH (+) | 12 | 12 | |
| 3p25.3–26.2 (D3S1038) | LOH (–) | 26 | 26 | 0.761 |
| | LOH (+) | 13 | 15 | |
| 1p (L-Myc) | LOH (–) | 28 | 27 | 0.169 |
| | LOH (+) | 7 | 14 | |
| 5q (APC, MCC) | LOH (–) | 29 | 19 | 0.006[a] |
| | LOH (+) | 4 | 14 | |
| 8p (LPL) | LOH (–) | 22 | 23 | 0.715 |
| | LOH (+) | 12 | 15 | |
| 9p (IFNA, D9S126) | LOH (–) | 25 | 27 | 0.699 |
| | LOH (+) | 14 | 18 | |
| 11p (H-ras, INS, RRM1, FSHB, WT1, CAT) | LOH (–) | 32 | 33 | 0.324 |
| | LOH (+) | 9 | 15 | |
| 13q (RB, D13S260) | LOH (–) | 32 | 34 | 0.869 |
| | LOH (+) | 14 | 16 | |
| 17q (NF1, NM23H1, D17S40, D17S21, D17S4) | LOH (–) | 27 | 26 | 0.641 |
| | LOH (+) | 17 | 20 | |
| 18q (DCC) | LOH (–) | 33 | 37 | 0.917 |
| | LOH (+) | 5 | 6 | |

[a]With multiple comparisons, Bonferroni's correction was used to set the level of significance at $P = 0.005$.

**Table 3** Correlation between Fhit expression and p53 abnormalities and mutations in the K-*ras* gene

| | p53 status | | | | | | K-*ras* codon 12 mutation | |
|---|---|---|---|---|---|---|---|---|
| | 17p13 (D17S5) LOH | | p53E5–8 SSCP | | p53 IHC | | | |
| | – | + | Wild-type | Shift | – | + | – | + |
| Fhit + | 20 | 13 | 38 | 9 | 27 | 19 | 38 | 9 |
| Fhit – | 10 | 21 | 37 | 15 | 24 | 28 | 50 | 2 |
| | *P* = 0.023 | | *P* = 0.261 | | *P* = 0.215 | | *P* = 0.016 | |

Our data add to the growing body of evidence that loss of Fhit expression is distinctly more frequent in lung cancers with squamous differentiation. In our experience, adenosquamous carcinomas lose Fhit expression to a similar degree as pure SCCs, whereas BACs have a Fhit negativity rate similar to conventional adenocarcinomas (Table 1). Two other immunohistochemical studies also showed that squamous carcinomas were significantly more likely to be Fhit-negative than adenocarcinomas (Sozzi et al, 1998; Tomizawa et al, 1998). In addition, it was previously shown that LOH at the *FHIT* locus is less common in adenocarcinomas compared to SCCs (Fong et al, 1997; Burke et al, 1998). Furthermore, *FHIT* abnormalities are very common in small-cell carcinomas but relatively infrequent in pulmonary carcinoids (Fong et al, 1997; Kovatich et al, 1998). These findings are confirmed by our observation that Fhit expression was lost in all three atypical carcinoids, but preserved in the single typical carcinoid tumour. The small number of SCLCs we stained for Fhit have all been negative (data not shown). In aggregate, the present and previously published data indicate that *FHIT* abnormalities are particularly common in those histological types of lung cancer (such as squamous and small-cell carcinomas) that are most closely associated with exposure to cigarette smoke.

In our series we looked for relationships between aberrant Fhit expression and a large number of other molecular genetic abnormalities in NSCLC. Loss of Fhit expression was correlated with LOH at 3p14.2, although 36% of cases were discordant (Table 2). The presence of Fhit immunoreactivity in LOH positive cases (14%) must reflect expression from the remaining 3p14.2 allele. The absence of Fhit staining in tumours without demonstrable LOH (22%) probably results from contaminating normal tissue in non-microdissected primary tumours subjected to molecular analysis. Similar findings were reported by others (Tomizawa et al, 1998). In addition, Sozzi et al have shown a good correlation between Fhit IHC and Southern, reverse transcription PCR (RT-PCR) and Western analyses (Sozzi et al, 1997b). The positive correlation of LOH at the *FHIT* locus and loss of Fhit expression is interesting because intragenic mutations do not appear to be a

*© 2000 Cancer Research Campaign*

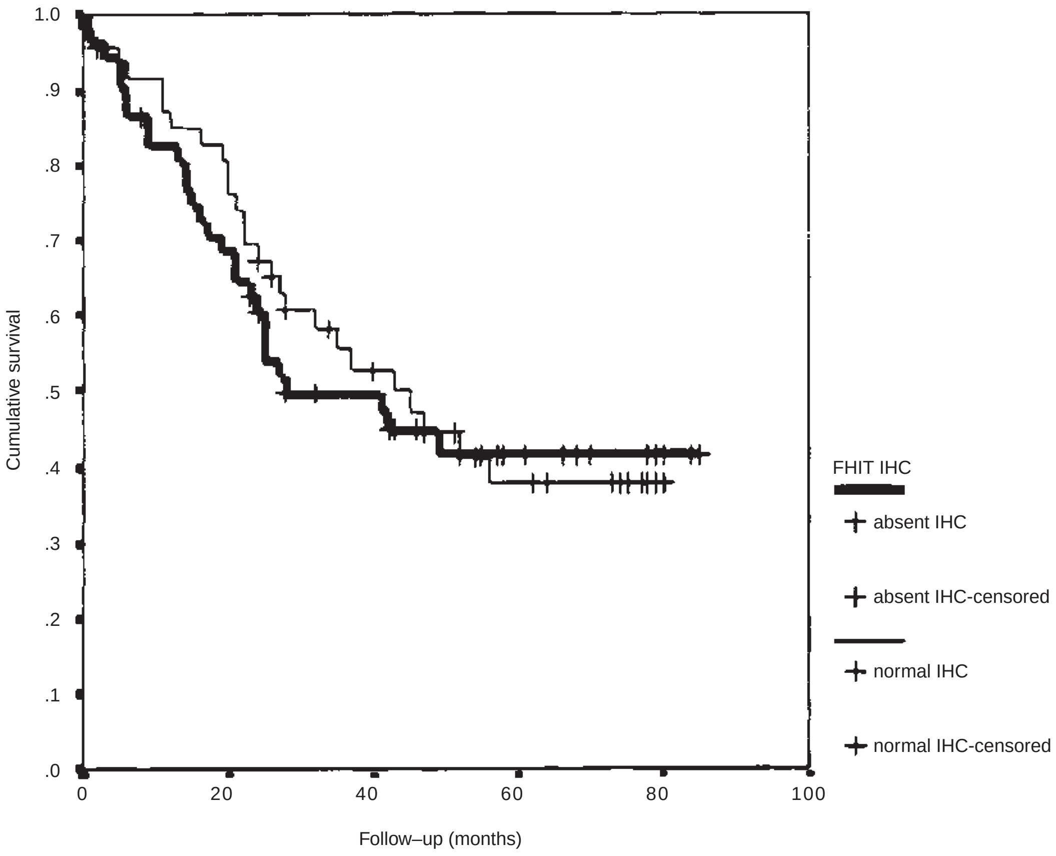

**Figure 2** Kaplan–Meier survival curve of the 99 cases of NSCLC stratified by Fhit immunohistochemical expression status. There was no significant difference in survival curves between those tumours with and those without Fhit expression common mechanism for inactivating the second allele. It is conceivable that loss of one *FHIT* allele may be associated with genetic or epigenetic promoter inactivation in the second allele, or with regulatory abnormalities upstream. In aggregate, these findings provide evidence that the *FHIT* gene is a critical and specific target in pulmonary carcinogenesis. In contrast, loss of Fhit expression was not associated with LOH at many other known or presumed tumour suppressor gene loci on 3p and on other chromosomal arms, with the possible exceptions of the APC/MCC locus on 5q and loci on 17p13 near TP53. In addition, we found no association between loss of Fhit staining and loss of IHC expression of RB and p16 or accumulation of p53. These findings suggest that disruption of *FHIT* function is independent of the other major known molecular abnormalities in lung cancer.

It was previously suggested that the genetic changes at the *FHIT* locus may be correlated with p53 mutations (Burke et al, 1998; Marchetti et al, 1998*b*), while other investigators found no statistically significant correlation between *FHIT* and p53 abnormalities in NSCLCs (Nelson et al, 1998). In our series, loss of Fhit expression was not correlated with p53 SSCP abnormalities in exons 5–8 or p53 accumulation by IHC (Table 3). Likewise, in the immunohistochemical study by Sozzi et al, Fhit down-regulation occurred at comparable frequencies in p53-positive and p53-negative NSCLCs (Sozzi et al, 1998).

Loss of Fhit expression was significantly less common in tumours harbouring a K-*ras* mutation (Table 3), suggesting that both events are not required in the great majority of NSCLCs. This observation is also in keeping with the selective occurrence of

**Table 4** Clinical parameters stratified by Fhit immunohistochemical expression

| | **Fhit immunohistochemistry** | | ***P*** |
|---|---|---|---|
| | **Positive** | **Negative** | |
| Mean age (years) | 62.4 | 59.7 | 0.221[a] |
| Pack-years | 36.5 | 48.1 | 0.071[a] |
| Sex | | | |
| Male | 29 | 41 | 0.061[b] |
| Female | 18 | 11 | |
| Smoking history | | | |
| Smoker | 42 | 49 | 0.472[c] |
| Non-smoker | 5 | 3 | |
| T stage | | | |
| 1 | 13 | 14 | |
| 2 | 31 | 32 | 0.670[b] |
| 3 | 3 | 6 | |
| N stage | | | |
| 0 | 28 | 34 | |
| 1 | 13 | 11 | 0.751[b] |
| 2 | 6 | 7 | |
| TNM stage | | | |
| I | 27 | 29 | |
| II | 11 | 11 | 0.883[b] |
| III | 9 | 12 | |

[a]*t*-test; [b] $\chi^2$ test; [c] Fisher's exact test.

K-*ras* mutations in adenocarcinomas and with the preferential targeting of *FHIT* in tumours with squamous differentiation. Interestingly, one of the two tumours showing both loss of Fhit and mutation of K-*ras* was an adenosquamous carcinoma. A lack of correlation between *FHIT* exon deletion and *ras* mutations was reported by Nelson et al (1998), and in heavy smokers, no significant association between *FHIT* LOH and K-*ras* abnormalities was demonstrable (Marchetti et al, 1998*b*).

In earlier work in our cohort of patients, and in two other series (Marchetti et al, 1998*a*; Tomizawa et al, 1998; Geradts et al, 1999), 3p14.2 LOH at or near the *FHIT* locus was not associated with impaired survival, while a fourth study (Burke et al, 1998) described an adverse effect on survival. We found that loss of Fhit protein expression was not significantly associated with a number of clinical parameters including age, gender and T, N and TNM stage, or survival (Table 4 and Figure 2). This is in agreement with the findings by Nelson et al (1998) and Sozzi et al (1998), both of whom also failed to detect a prognostic effect of aberrant Fhit expression in resected NSCLCs. In contrast, Tomizawa et al (1998) found loss of Fhit protein expression to be associated with adverse survival specifically in stage I NSCLCs. However, tumours with an intermediate level of Fhit reactivity were excluded from the survival analysis. Thus, three of four studies are consistent with a role of *FHIT* abnormalities at a relatively early stage of pulmonary neoplasia, rather than in the metastatic progression of invasive lung cancers.

It has been suggested that the *FHIT* gene is specifically targeted by carcinogens in cigarette smoke, and a number of reports described an increased frequency of *FHIT* abnormalities at the DNA or protein level in smokers (Sozzi et al, 1997*a*, 1998; Marchetti et al, 1998*b*; Nelson et al, 1998; Tomizawa et al, 1998). Our series of 99 NSCLC patients included only eight non-smokers, which may in part account for the lack of a statistically significant correlation between smoking history and Fhit expression (Table 4) or LOH at the *FHIT* locus (Geradts et al, 1999). However, we did find a tendency for an increase in loss of Fhit expression with extensive cigarette consumption ($P = 0.07$).

In summary, our immunohistochemical analysis of Fhit expression in 99 well-characterized NSCLCs confirms that loss of Fhit protein expression is among the most common molecular abnormalities in this tumour type. The positive correlation with 3p14.2 LOH further suggests that the *FHIT* gene is an important target for disruption at the DNA and cDNA levels in bronchogenic carcinomas. The lack of association between loss of Fhit expression and multiple other molecular abnormalities emphasizes the independent nature of disrupting the Fhit pathway in lung carcinogenesis. The particularly frequent loss of expression in tumours with squamous differentiation and the inverse correlation with K-*ras* mutations provides additional evidence that different histological lung cancer types are associated with particular molecular alterations. Finally, Fhit down-regulation was not correlated with important clinical parameters including stage and survival suggesting that it plays a role early in the pathogenesis of lung cancer rather than in later metastatic stages.

### ACKNOWLEDGEMENTS

We thank Dr Kay Huebner for providing the anti-Fhit polyclonal antibody and Rob Maynard for performing the immunohistochemical assays. This work was supported by grants from the Queensland Cancer Fund, the National Health and Medical Research Council of Australia, and the National Cancer Institute (USA) ROI CA 71618 and Lung Cancer SPORE Grant P50 CA 70907.

### REFERENCES

Baffa R, Veronese ML, Santoro R, Mandes B, Palazzo JP, Rugge M, Santoro E, Croce CM and Huebner K (1998) Loss of *FHIT* expression in gastric carcinoma. *Cancer Res* **58**: 4708–4714

Burke L, Khan MA, Freedman AN, Gemma A, Rusin M, Guinee DG, Bennett WP, Caporaso NE, Fleming MV, Travis WD, Colby TV, Trastek V, Pairolero PC, Tazelaar HD, Midthun DE, Liotta LA and Harris CC (1998) Allelic deletion analysis of the *FHIT* gene predicts poor survival in non-small cell lung cancer. *Cancer Res* **58**: 2533–2536

Fong KM, Zimmerman PV and Smith PJ (1994) Correlation of loss of heterozygosity at 11p with tumour progression and survival in non-small cell lung cancer. *Genes Chromosomes Cancer* **10**: 183–189

Fong KM, Kida Y, Zimmerman PV, Ikenaga M and Smith PJ (1995*a*) Loss of heterozygosity frequently affects chromosome 17q in non-small cell lung cancer. *Cancer Res* **55**: 4268–4272

Fong KM, Zimmerman PV and J SP (1995*b*) Tumor progression and loss of heterozygosity at 5q and 18q in non-small cell lung cancer. *Cancer Res* **55**: 220–223

Fong KM, Zimmerman PV and Smith PJ (1995*c*) Microsatellite instability and other molecular abnormalities in non-small cell lung cancer. *Cancer Res* **55**: 28–30

Fong KM, Biesterveld EJ, Virmani A, Wistuba I, Sekido Y, Bader SA, Ahmadian M, Ong ST, Rassool FV, Zimmerman PV, Giaccone G, Gazdar AF and Minna JD (1997) *FHIT* and FRA3B 3p14.2 allele loss are common in lung cancer and preneoplastic bronchial lesions and are associated with cancer-related *FHIT* cDNA splicing aberrations. *Cancer Res* **57**: 2256–2267

Fong KM, Zimmerman PV and Smith PJ (1998) KRAS codon 12 mutations in Australian non-small cell lung cancer. *Aust NZJ Med* **28**: 184–189

Geradts J, Fong KM, Zimmerman PV, Maynard R and Minna JD (1999) Correlation of abnormalities of RB, p16$^{ink4a}$, and p53 expression with 3p loss of heterozygosity, other genetic abnormalities and clinical features in 103 primary non-small cell lung cancers. *Clin Cancer Res* 5: 791–800

Greenspan DL, Connolly DC, Wu R, Lei RY, Vogelstein JTC, Kim Y-T, Mok JE, Munoz N, Bosch FX, Shah K and Cho KR (1997) Loss of *FHIT* expression in cervical carcinoma cell lines and primary tumors. *Cancer Res* **57**: 4692–4698

Hadaczek P, Siprashvili Z, Markiewski M, Domagala W, Druck T, McCue PA, Pekarsky Y, Ohta M, Huebner K and Lubinski J (1998) Absence or reduction of Fhit expression in most clear cell renal carcinomas. *Cancer Res* **58**: 2946–2951

Kovatich A, Friedland DM, Druck T, Hadaczek P, Huebner K, Comis RL, Hauck W and McCue PA (1998) Molecular alterations to human chromosome 3p loci in neuroendocrine lung tumors. *Cancer* **83**: 1109–1117

Marchetti A, Pellegrini S, Bertacca G, Buttitta F, Gaeta P, Carnicelli V, Nardini V, Griseri P, Chella A, Angeletti CA and Bevilacqua G (1998*a*) *FHIT* and *p53* gene abnormalities in bronchioloalveolar carcinomas. Correlations with clinicopathological data and K-*ras* mutations. *J Pathol* **184**: 240–246

Marchetti A, Pellegrini S, Sozzi G, Bertacca G, Gaeta P, Buttitta F, Carnicelli V, Griseri P, Chella A, Angeletti CA, Pierotti M and Bevilacqua G (1998*b*) Genetic analysis of lung tumours of non-smoking subjects: p53 gene mutations are constantly associated with loss of heterozygosity at the FHIT locus. *Br J Cancer* **78**: 73–78

Nelson HH, Wiencke JK, Gunn L, Wain JC, Christiani DC and Kelsey KT (1998) Chromosome 3p14 alterations in lung cancer: evidence that *FHIT* exon deletion is a target of tobacco carcinogens and asbestos. *Cancer Res* **58**: 1804–1807

Ohta M, Inoue H, Cotticelli MG, Kastury K, Baffa R, Palazzo J, Siprashvili Z, Mori M, McCue P, Druck T, Croce CM and Huebner K (1996) The *FHIT* gene, spanning the chromosome 3p14.2 fragile site and renal carcinoma-associated t(3;8) breakpoint, is abnormal in digestive tract cancers. *Cell* **84**: 587–597

Otterson GA, Xiao GH, Geradts J, Jin F, Chen W, Niklinska W, Kaye FJ and Yeung RS (1998) Protein expression and functional analysis of the *FHIT* gene in human tumor cells. *J Natl Cancer Inst* **90**: 426–432

Simon B, Bartsch D, Barth P, Prasnikar N, Munch K, Blum A, Arnold R and Goke B (1998) Frequent abnormalities of the putative tumor suppressor gene *FHIT* at 3p14.2 in pancreatic carcinoma cell lines. *Cancer Res* **58**: 1583–1587

Siprashvili Z, Sozzi G, Barnes LD, McCue P, Robinson AK, Eryomin V, Sard L, Tagliabue E, Greco A, Fusetti L, Schwartz G, Pierotti MA, Croce CM and Huebner K (1997) Replacement of FHIT in cancer cells suppresses tumorigenicity. *Proc Natl Acad Sci USA* **94**: 13771–13776

*© 2000 Cancer Research Campaign*

Sozzi G, Veronese ML, Negrini M, Baffa R, Cotticelli MG, Inoue H, Tornielli S, Pilotti S, De Gregorio L, Pastorino U, Pierotti MA, Ohta M, Huebner K and Croce CM (1996) The *FHIT* gene at 3p14.2 is abnormal in lung cancer. *Cell* 85: 17–26

Sozzi G, Sard L, De Gregorio L, Marchetti A, Musso K, Buttitta F, Tornielli S, Pellegrini S, Veronese ML, Manenti G, Incarbone M, Chella A, Angeletti CA, Pastorino U, Huebner K, Bevilaqua G, Pilotti S, Croce CM and Pierotti MA (1997*a*) Association between cigarette smoking and *FHIT* gene alterations in lung cancer. *Cancer Res* 57: 2121–2123

Sozzi G, Tornielli S, Tagliabue E, Sard L, Pezzella F, Pastorino U, Minoletti F, Pilotti S, Ratcliffe C, Veronese ML, Goldstraw P, Huebner K, Croce CM and Pierotti MA (1997*b*) Absence of Fhit protein in primary lung tumors and cell lines with *FHIT* gene abnormalities. *Cancer Res* 57: 5207–5212

Sozzi G, Pastorino U, Moiraghi L, Tagliabue E, Pezzella F, Ghirelli C, Tornielli S, Sard L, Huebner K, Pierotti MA, Croce CM and Pilotti S (1998) Loss of *FHIT* function in lung cancer and preinvasive bronchial lesions. *Cancer Res* 58: 5032–5037

Tomizawa Y, Nakajima T, Kohno T, Saito R, Yamaguchi N and Yokota J (1998) Clinicopathological significance of Fhit protein expression in stage I non-small cell lung cancer. *Cancer Res* 58: 5478–5483

© 2000 Cancer Research Campaign 
